# Genome Engineering of Primary and Pluripotent Stem Cell-Derived Hepatocytes for Modeling Liver Tumor Formation

**DOI:** 10.3390/biology13090684

**Published:** 2024-09-02

**Authors:** Lulu Zhang, Xunting Wang, Xuelian Yang, Yijia Chi, Yihang Chu, Yi Zhang, Yufan Gong, Fei Wang, Qian Zhao, Dongxin Zhao

**Affiliations:** 1 Shanghai Institute of Materia Medica, Chinese Academy of Sciences, Shanghai 201203, China; zhanglulu@simm.ac.cn (L.Z.); chiyijia@simm.ac.cn (Y.C.); chuyh2023@shanghaitech.edu.cn (Y.C.); yf258046@163.com (Y.G.); wangfei2@simm.ac.cn (F.W.); zhaoqian@simm.ac.cn (Q.Z.); 2 University of Chinese Academy of Sciences, Beijing 101408, China; 3 School of Chinese Materia Medica, Nanjing University of Chinese Medicine, Nanjing 210023, China; wangxunting@simm.ac.cn (X.W.); yangxuelian@simm.ac.cn (X.Y.); zhangyi3@simm.ac.cn (Y.Z.); 4 School of Life Science and Technology, Shanghai Tech University, Shanghai 201210, China

**Keywords:** hepatocytes, organoids, human pluripotent stem cells, CRISPR-Cas9, genome editing

## Abstract

**Simple Summary:**

Primary hepatocytes constitute the majority of liver cells and are responsible for many liver-related diseases, including cancer. However, the lack of robust methods for engineering primary hepatocytes ex vivo has hindered their versatile application in disease modeling. Here, we worked to enable genome manipulation in primary mouse hepatocytes in both monolayer culture and organoids, and achieved up to 80% and 20% single gene knockout efficiency, respectively. Using genome engineering of human pluripotent stem cell-derived hepatocytes, we developed a spectrum of isogenic cellular models of hepatocarcinoma with various single or combined oncogenic alterations, and demonstrated that the introduction of these alterations initiated malignant transformation, accelerated cancer growth, and altered transcriptomic profiles and pathway activities. These results reveal that oncogenic alterations dominate cancer characteristics and highlight a powerful genome-engineering-based platform for cancer mechanism studies.

**Abstract:**

Genome editing has demonstrated its utility in generating isogenic cell-based disease models, enabling the precise introduction of genetic alterations into wild-type cells to mimic disease phenotypes and explore underlying mechanisms. However, its application in liver-related diseases has been limited by challenges in genetic modification of mature hepatocytes in a dish. Here, we conducted a systematic comparison of various methods for primary hepatocyte culture and gene delivery to achieve robust genome editing of hepatocytes ex vivo. Our efforts yielded editing efficiencies of up to 80% in primary murine hepatocytes cultured in monolayer and 20% in organoids. To model human hepatic tumorigenesis, we utilized hepatocytes differentiated from human pluripotent stem cells (hPSCs) as an alternative human hepatocyte source. We developed a series of cellular models by introducing various single or combined oncogenic alterations into hPSC-derived hepatocytes. Our findings demonstrated that distinct mutational patterns led to phenotypic variances, affecting both overgrowth and transcriptional profiles. Notably, we discovered that the PI3KCA E542K mutant, whether alone or in combination with exogenous c-MYC, significantly impaired hepatocyte functions and facilitated cancer metabolic reprogramming, highlighting the critical roles of these frequently mutated genes in driving liver neoplasia. In conclusion, our study demonstrates genome-engineered hepatocytes as valuable cellular models of hepatocarcinoma, providing insights into early tumorigenesis mechanisms.

## 1. Introduction

Genome editing technology has revolutionized cell therapy and basic biomedical research, offering powerful tools to precisely manipulate genomic DNA and alter cellular characteristics and functions [1]. In addition to the compelling therapeutic applications, CRISPR/Cas (clustered regularly interspaced short palindromic repeats/CRISPR-associated proteins)-mediated genome editing has significantly facilitated the generation of more accurate genetically altered disease models, which enable the precise recapitulation of pathogenesis and disease developmental processes. 

Genetically modified cellular models hold great promise for providing deeper insights into disease mechanisms and the impact of genetic variants. For example, tumorigenesis has been recapitulated using CRISPR-mediated cellular models for many types of cancer, including brain [2], breast [3], colon [4,5], pancreas [6], stomach [7], etc. These models have been widely used to elucidate the roles of oncogenic genes, mutational signatures, and drug response. Beyond cancer, genetically modified cellular models have been created for studying congenital diarrheal disorder [8], cystic fibrosis [9,10], and severe acute respiratory syndrome coronavirus 2 (SARS-CoV-2) [11,12]. These advances highlight the incredible value of modeling human disease by genetically manipulating cells resembling their tissue of origin. 

The liver is a major organ responsible for detoxification, metabolism, and various biochemical processes in the human body [13]. Dysregulation of liver functions leads to complex chronic diseases, such as hepatocellular carcinoma and nonalcoholic fatty liver disease. The first CRISPR-mediated genome editing in mouse liver used hydrodynamic injection to deliver DNA plasmids expressing Cas9 and single guide RNAs (sgRNAs) to the liver [14]. Subsequently, other delivery methods, including adeno-associated virus (AAV) and nanoparticles, have also been employed for direct in vivo genome editing in animal models [15].

Despite substantial progress in genome editing of hepatocytes in vivo, efficient genome editing methods for primary hepatocytes in vitro remain underdeveloped, due to challenges in maintaining long-term cultures of functional hepatocytes [16,17,18]. To date, all efforts to extend the culture of primary hepatocytes in monolayer have faced limitations, such as species-specific differences hindering relevance to mouse hepatocytes or dedifferentiation processes impairing hepatocyte functions. In recent years, methods for culturing primary hepatocyte organoids derived from either adult or fetal liver have been described [19,20], showing promise for applications in disease modeling, gene and cell therapy, and drug screening. However, expanding adult primary hepatocyte organoids, unlike their fetal liver-derived counterparts, remains challenging and may limit their potential utility [21]. Therefore, benchmarking the efficiency and robustness of current strategies for the construction of genetically modified cellular models of liver diseases is crucial. This allows assessing the applicability of these models and benefits further research in developing better technologies.

In this study, we systematically compared methods for the culturing and genome editing of murine primary hepatocytes in vitro. The genome editing efficiency in primary adult hepatocytes reached up to 80% in monolayer and 20% in organoids, respectively. In comparison, we performed genome editing in human pluripotent stem cell-derived hepatocytes, which enabled long-term culture with high editing efficiency. By introducing alterations in tumor-associated genes in hepatocytes, we established an in vitro model system to recapitulate liver tumorigenesis. Furthermore, we demonstrated that various oncogenic genes drive the acquisition of various malignant features, providing a platform for investigating gene functions during the onset of liver cancers. This model system offers valuable insights into liver cancer development and can be used to test potential therapeutic interventions.

## 2. Materials and Methods

### 2.1. Materials and Reagents

William’s E Medium, B27 (serum free), B27 (minus vitamin A), N2, Advanced DMEM/F12, insulin-transferrin-selenium (ITS), and fetal bovine serum were purchased from Gibco, Thermo Fisher Scientific (Waltham, MA, USA). Bovine Serum Albumin (BSA), Liberase^TM^, N-acetylcysteine, Human [Leu15]-gastrin I, CHIR99021, and Nicotinamide were obtained from Sigma Aldrich, Merck KGaA (Darmstadt, Germany). Forskolin, SB431542, IWP2, DAPT, LDN-193189, Y-27632, and A83-01 were purchased from Selleck (Shanghai, China). Human recombinant epidermal growth factor (EGF), recombinant human FGF7, recombinant human FGF10, recombinant human HGF, and Activin a were obtained from Peprotech (Rocky Hill, NJ, USA). Recombinant Human Wnt-3a Protein was obtained from R&D systems (Minneapolis, MN, USA). The 96-well Ultra-Low-Attachment plate, growth factors-reduced Matrigel for organoids culture (#354230), and Matrigel for human pluripotent stem cell-coated (#354277) were purchased from Corning (Corning, NY, USA). Pluripotency Growth Master 1 (PGM1) human pluripotent stem cell culture medium was purchased from Cellapy (Beijing, China).

### 2.2. Primary Mouse Hepatocytes Isolation

All animal experiments were performed according to the institutional animal care ethics guidelines and approved by the Institutional Animal Care and Use Committee (IACUC) of Shanghai Institute of Materia Medica, Chinese Academy of Sciences. Mouse hepatocytes were isolated from transgenic Cas9-EGFP (Jackson Laboratory, #026175, Bar Harbor, ME, USA) mice using a two-step collagenase perfusion method, as previously described [22]. Briefly, after successfully anesthetizing the mice, a “U” incision was made in the lower abdomen to expose the liver, portal vein, and inferior vena cava. A needle was then inserted into the vena cava, the portal vein was cut, and the liver was perfused at 3 mL/min with pre-warmed perfusion buffer for 10 min to clear blood. Following this, perfusion was continued with pre-warmed digestion buffer containing 25 μg/mL Liberase and Ca^2+^ at 3 mL/min for 3 min. After dissociation, the cells were filtered through a 70 μm filter. Hepatocytes were further separated and purified by Percoll gradient centrifugation at low speed. The hepatocytes were then incubated in DMEM (low glucose) with 5% FBS for at least 3 h. The plating medium was then aspirated, and each well was gently rinsed twice with PBS to remove unadhered cells. The maintenance medium was changed every 2 days.

The primary hepatocyte maintenance media were as follows: Williams’ Medium E with supplements (WEM), Williams’ medium E containing 1× B27, 1× Glutamax, and 1× penicillin-streptomycin; 5C condition (5C): WEM supplemented with Forskolin (20 μM), SB431542 (10 μM), IWP2 (0.5 μM), DAPT (5 μM), and LDN193189 (0.1 μM); Hepatocyte Culture Medium (HCM): manufactured by Lonza (CC3198); Proliferation human hepatocytes condition (ProliHH): Advanced DMEM/F12 supplemented with 1 mM N-acetylcysteine, 1× B27 (minus vitamin A), 1× N2 supplement, 10 nM gastrin I, 25 μg/mL recombinant Human Wnt-3a Protein, 50 ng/mL EGF, 2 ng/mL FGF10, 25 ng/mL recombinant human HGF, 25 ng/mL human recombinant bFGF, 5 μM A83-01, 10 μM Y-27632, 1% penicillin-streptomycin, and 1% fetal bovine serum.

### 2.3. Cell Viability Assay

As mentioned above, primary mouse hepatocytes were plated in Collagen I-coated 96-well white/transparent bottom plates at a density of 1.5 × 10^5^ cells/cm^2^. 

Cell viability was quantified on days 1, 4, 7, and 10 using a CellTiter-Meiluncell Luminescent Cell Viability Assay Kit (Meilunbio, Dalian, China). Briefly, 100 μL of CellTiter-Meiluncell reagent was added to each well, incubated with cells for 10 min at room temperature, and then measured using a PE EnVision^®^ Multi-Label Plate Reader (PerkinElmer, Waltham, MA, USA). All experiments were performed independently in triplicate. 

### 2.4. Primary Mouse Hepatocytes Organoids Culture

The dome-cultured organoids were established from freshly isolated primary mouse hepatocytes, as previously described [20]. Briefly, approximately 20,000 hepatocytes were mixed with 30 μL Matrigel and then seeded into a well of a 24-well plate. After the Matrigel had solidified, organoid culture medium was added. The organoid culture medium consisted of AdDMEM/F12 (with HEPES, GlutaMax and Penicillin-Streptomycin) plus 15% RSPO1 conditioned medium (home-made), B27 (minus vitamin A), 50 ng/mL EGF, 1.25 mM N-acetylcysteine, 10 nM Human [Leu15]-gastrin I, 3 mM CHIR99021, 25 ng/mL HGF, 50 ng/mL FGF7, 50 ng/mL FGF10, 1 mM A83-01, 10 mM Nicotinamide, and 10 μM Y-27632. 

For floating culture, approximately 10,000 freshly isolated hepatocytes were suspended in 100 μL organoid culture medium supplemented with 5% Matrigel, and then cultured in a 96-well ultra-low-attachment plate. The medium was refreshed every 4 days. 

### 2.5. Immunofluorescence Staining

Immunofluorescence staining was performed according to a previously established protocol [23]. Briefly, the cell culture medium was discarded, and the cells were fixed with 4% paraformaldehyde (pH 7.4) for 15 min at room temperature, followed by washing with PBS. The cells were then incubated in blocking buffer (PBS containing 5% normal donkey serum and 0.1% Triton X-100) at room temperature for 60 min. After washing with PBS, the cells were incubated overnight at 4 °C with primary antibodies diluted in blocking buffer at the recommended concentrations (CYP3A4, Proteintech, Chicago, IL, USA, 1:600; Albumin, Bethyl, Montgomery, TX, USA, 1:2000). The following day, the primary antibody solution was discarded, and the cells were washed with PBS, then incubated with fluorescent dye-labeled secondary antibodies (Invitrogen, Thermo Fisher Scientific, Waltham, MA, USA, 1:2000) diluted in PBS containing 1% BSA for 60 min at room temperature, protected from light. After discarding the secondary antibody solution and washing with PBS, the cell nuclei were stained with PBS containing 1 μg/mL DAPI for 5 min at room temperature, also protected from light. Finally, after discarding the DAPI solution and washing with PBS, the cells were visualized using an Olympus IX73 fluorescence microscope. For whole-mount immunofluorescence of 3D organoids, the organoids were collected and incubated in cold PBS containing 0.1% BSA to dissolve the Matrigel. All subsequent procedures were performed as described for monolayer cells.

### 2.6. Electroporation 

Electroporation was performed using a P3 Primary Cell 4D-Nucleofector™ X Kit (Lonza, Basel, Switzerland). According to the manufacturer’s instructions, 600,000 freshly isolated mouse hepatocytes were resuspended in 100 μL of Lonza electroporation buffer P3 (82 μL Nucleofector Solution and 18 μL Supplement) containing 5 μg of DNA. The cell suspension was then transferred into Nucelocuvette^TM^ vessels and electroporated using a Lonza 4D-Nucleofector^TM^ core unit with the DS-150 program. After electroporation, the cells were resuspended in plating medium and seeded onto collagen I-coated plates for at least 3 h. The plating medium was then replaced with pre-warmed HCM. After 72 h, images were captured using an Olympus IX73 fluorescence microscope and the transfection efficiency was quantified using Image J software (version 1.53e).

### 2.7. Lipofection

Lipofectamine™ 3000 (Invitrogen, Thermo Fisher Scientific) was used for lipofection, according to the manufacturer’s instructions. For each well of a 24-well plate, 1 μL of Lipofectamine 3000 reagent was diluted in 25 μL of Opti-MEM (Gibco, Thermo Fisher Scientific). Separately, 0.5 μg of DNA and 1 μL of P3000 reagent were added to 25 μL of Opti-MEM in another tube. The DNA solution and the Lipofectamine 3000 reagent solution were then combined and incubated at room temperature for 15 min. After incubation, a 50 μL mixture of DNA-Lipofectamine complex was added to the cells, ensuring even distribution across the well by gently swirling the plate. Twelve hours later, the medium was replaced with fresh HCM. Then, 72 h after lipofection, images were captured using an Olympus IX73 fluorescence microscope, and transfection efficiency was quantified using ImageJ software (version 1.53e).

### 2.8. AAV Production and Infection 

We constructed an AAV vector expressing the EGFP reporter under the control of the EF1a promoter, and verified the accuracy of its DNA sequences by Sanger sequencing. For AAV production, the AAV vector, rep/cap packaging plasmids, and adenoviral helper plasmids were co-transfected into HEK293T cells. After 72 h of incubation, both the cell pellet and supernatant were collected. The cell pellet was lysed through 3 freeze–thaw cycles, and the combined cell lysate and supernatant were concentrated by precipitation in 10% PEG 8000 and 1.0 M NaCl at 4 °C overnight. The precipitate was then resuspended in PBS buffer with a final concentration of 200U/mL Benzonase (Millipore, Merck KGaA, Darmstadt, Germany). Four discontinuous iodixanol gradient solutions (15%, 25%, 40%, and 60%) were prepared in a Beckman tube. The virus-containing supernatant was layered onto the top of the gradient and centrifuged at 58,000 rpm in a Beckman Type 70Ti rotor for 2 h at 18 °C. The 40% iodixanol fraction containing AAVs was collected and the purified virus was further concentrated via buffer exchange into PBS with 0.001% F68 (Gibco, Thermo Fisher Scientific). 

The titers of AAVs were measured by qPCR using SYBR Green, with the primers targeting the AAV2 ITR regions: forward ITR primer, 5′-GGAACCCCTAGTGATGGAGTT; reverse ITR primer, 5′-CGGCCTCAGTGAGCGA. The quality of the AAV was confirmed through viral infection assays in cell lines such as HEK293T and Hepa1-6.

For AAV infection, freshly isolated mouse hepatocytes were resuspended in plating medium and seeded onto collagen I-coated plates for at least 3 h. Following this, the plating medium was replaced with pre-warmed HCM. High-titer AAVs (approximately MOI of 30) were added to the HCM, along with polybrene at a final concentration of 8 μg/mL. 12 h later, the medium was replaced with fresh HCM. After 72 h, images were captured using an Olympus IX73 fluorescence microscope, and transfection efficiency was quantified using ImageJ software (version 1.53e). 

### 2.9. Lentivirus Production and Infection

For lentivirus production, 6 μg pMD2.G, 24 μg psPAX2, and 18 μg of lentiviral vector were co-transfected in one 15 cm dish of HEK293T cells using 120 μL PEI. After 12 h, the media was changed for fresh culture media. Viral particles were collected at 48 h and 72 h after transfection, respectively, then concentrated with Millipore 100,000 NMWL centrifugal ultrafilters, divided into aliquots and frozen at −80 °C.

The infection process and the multiplicity of infection of lentivirus was the same as for AAV.

### 2.10. Cloning of Exogenous Oncogenes Plasmids 

The PIK3CA E542K and MYC open reading frame (ORF) sequences were cloned into a lentiviral vector derived from EF1a_mCherry_P2A_Hygro (Addgene, #135003, Cambridge, MA, USA). In this modified vector, the mCherry was replaced with a puromycin resistance gene, and the hygromycin resistance gene was replaced with the ORF. The accuracy of the DNA sequences of each plasmid was confirmed using Sanger sequencing. 

### 2.11. sgRNA Selection and Cloning

We initially selected two sgRNA sequences per gene from the genome-wide CRISPR screen library (Brunello, Addgene, #73179), which was designed using optimized metrics that combine improved on-target activity predictions (Rule Set 2) with an off-target score, the cutting frequency determination [24]; we then validated the high knockout efficiency of each sgRNA in HEK293T cells during preliminary experiments, and the sgRNA sequences we eventually used are shown in Table 1.

The selected sgRNAs were cloned into the lentiviral vector CROP-seq-EGFP, a modified version of CROP-seq-opti (Addgene, #106280), where the puromycin resistance gene was replaced with an EGFP reporter. The empty vector was initially digested with the restriction enzyme BsmBI and then recombined with a dsDNA fragment containing the sgRNA sequences using Gibson Assembly. The accuracy of the DNA sequences of each plasmid was confirmed using Sanger sequencing.

### 2.12. Cell Differentiation and Genetic Modification

The H1 human embryonic stem cells were obtained from WiCell and cultured in Pluripotency Growth Master 1 (PGM1) human pluripotent stem cell culture medium (Cellapy). H1 cells were seeded at a density of 5 × 10^4^ cells/cm^2^ in Matrigel-coated 48-well plates and maintained in complete PGM1 culture medium for 1–2 days. To generate definitive endoderm, H1 cells were cultured in RPMI1640 medium supplemented with 0.5 mg/mL albumin fraction V and 100 ng/mL Activin a for 1 day. Over the next 2 days, 0.1% and 1% insulin-transferrin-selenium (ITS) were added to the DAY1 medium, respectively. Subsequent steps for the sequential differentiation of the definitive endoderm into posterior foregut, hepatic progenitors, and hepatocytes stages were carried out according to a previously established protocol [25].

We conducted lentivirus-based genetic modification of hPSC-derived hepatocytes at the hepatic progenitor stage, when the cells exhibited high proliferative capacity and transfection efficiency. To ensure high modification efficiency, we enriched the sgRNA-expressing cells through fluorescence-activated cell sorting (FACS) and the ORF-expressing cells through puromycin selection. After eliminating uninfected cells, the remaining cells continued through differentiation to produce mature hepatocytes for subsequent functional analysis experiments.

### 2.13. Cell Proliferation Quantification Assay

All operations were carried out according to the recommendations of the BeyoClick™ EdU-594 kit. Briefly, 20 μM EdU solution was prepared by diluting it in the culture medium. When the genetic modified hepatocytes reached 80% confluence, 20 μM EdU solution was used to replace a half of the culture media in the plates, and the cells were incubated for 2 h. After labeling with EdU, the cells were digested and fixed with 4% paraformaldehyde at room temperature for 15 min. Following fixation, cells were permeabilized with 0.3% Triton X-100 in PBS for 10 min. The click additive solution was then prepared according to the manufacturer’s instructions, and cells were incubated with the click additive solution at room temperature, protected from light, for 30 min. EdU quantification was performed by flow cytometry.

### 2.14. RNA Sequencing

RNA-sequencing libraries were prepared based on the smart-seq3 and smart-seq3xpress method [26,27]. First, ASGPR (hepatic surface marker) and EGFP (sgRNA lentivirus infected) double-positive cells were enriched by fluorescence-activated cell sorting (FACS). The sorted cells were then resuspended in 15 µL of lysis buffer and incubated for 10 min at 72 °C in a thermal cycler, followed by immediate cooling on ice. The lysis buffer contained 0.5 U µL^−1^ of recombinant RNase inhibitor (RRI) (Takara, Shiga, Japan), 0.15% Triton X-100 (Sigma Aldrich, Merck KGaA), 0.5 mM dNTP (Thermo Fisher Scientific), 1 µM Smart-seq3 oligo-dT primer (5′-biotin-ACGAGCATCAGCAGCATACGA T30VN-3′; GENEWIZ, Suzhou, China), and 5% PEG (Sigma Aldrich, Merck KGaA). Reverse transcription (RT) was performed by the addition of 5 µL RT mix, containing 25 mM Tris-HCl (pH 8.0, Sigma Aldrich, Merck KGaA), 30 mM NaCl (Invitrogen, Thermo Fisher Scientific), 2.5 mM MgCl_2_ (Invitrogen, Thermo Fisher Scientific), 1 mM GTP (Invitrogen, Thermo Fisher Scientific), 8 mM DTT (Thermo Fisher Scientific), 0.5 U µL^−1^ RRI, 2 µM of improved TSOs (improved TSOs; 5′-Biotin-AGAGACAGATTGCGCAATGNNNNNNNNWWrGrGrG-3′; GENEWIZ), and 2 U µL^−1^ of Maxima H-minus reverse transcriptase enzyme (Thermo Fisher Scientific). The reaction was carried out at 42 °C for 90 min, followed by 10 cycles of 2 min at 50 °C and 2 min at 42 °C. The reaction was then terminated by incubating at 85 °C for 5 min. cDNA amplification was performed by adding 30 µL of PCR mix, containing 1x KAPA HiFi HotStart ReadyMix (Roche, Basel, Switzerland), 0.5 µM Smartseq3 forward PCR primer (5′-TCGTCGGCAGCGTCAGATGTGTATAAGAGACAGATTGCGCAATG-3′; GENEWIZ), and 0.5 µM Smartseq3 reverse PCR primer (5′-ACGAGCATCAGCAGCATACGA-3′; GENEWIZ).

After PCR amplification, the cDNA was purified using a 0.6:1 volume of VAHTS DNA Clean Beads (Vazyme, Nanjing, China). The purified cDNA was analyzed on an Agilent 4150 Bioanalyzer to assess cDNA size distribution, and its concentration was measured on a Qubit 3.0 Fluorometer (Invitrogen, Thermo Fisher Scientific) with a Qubit dsDNA High Sensitivity Assay kit (Invitrogen, Thermo Fisher Scientific). The cDNA was then diluted to 200–500 pg µL^−1^. For tagmentation using our home-made Tn5, 50 ng of cDNA in 10 µL water was mixed with 40 µL of tagmentation mix, which consisted of 5 µL of Tn5, 25 µL of 2× TD buffer (25 mM Tris-HCl, pH 7.5, 10 mM MgCl_2_, 20% DMF), and 10 µL of water. The mixture was incubated at 55 °C for 5 min in a thermal cycler. Tagmented samples were then purified with 0.8:1 volume of VAHTS DNA Clean Beads.

Sequence library amplification was performed using 1.25 µL of custom-designed Nextera index primers (0.5 µM each) containing 8 bp indexes and 12.5 µL of NEBNext^®^ High-Fidelity 2X PCR Master Mix (NEB, Ipswich, MA, USA). The samples were purified with a 0.7:1 volume of VAHTS DNA Clean Beads, analyzed on an Agilent 4150 Bioanalyser, and DNA concentration was measured using a Qubit 3.0 Fluorometer. The libraries were sequenced on a NovaSeq S4 flow cell with a 150 bp paired end. 

### 2.15. RNA Sequencing Data Analysis

Sequencing reads from SMART-seq3 were processed using UMI-tools (version 1.1.4) to extract UMI-containing reads. The reads were then aligned to the GRCh38 human reference genome (Gencode v43) using STAR (version 2.7.11a). UMI reads were assigned to exon features using featureCounts (version 2.0.3). Normalization and differential expression analysis of the counts were performed using the Deseq2 R package (version 1.2.4). 

Differences in gene set variation analysis (GSVA) scores, generated by the GSVA R package, were evaluated using the limma R package. 

Weighted gene co-expression network analysis (WGCNA) was conducted using the WGCNA R package. Genes within each identified co-expression module were grouped based on their expression correlation with the entire module. Gene ontology (GO) and Kyoto Encyclopedia of Genes and Genomes (KEGG) pathway enrichment analysis were performed using the clusterProfiler R package. The co-expression network was visualized using Cytoscape software (v3.10.2). The accession number for the raw and analyzed data reported in this paper is GSE275241.

### 2.16. Statistical Analysis

**Figure 1:**
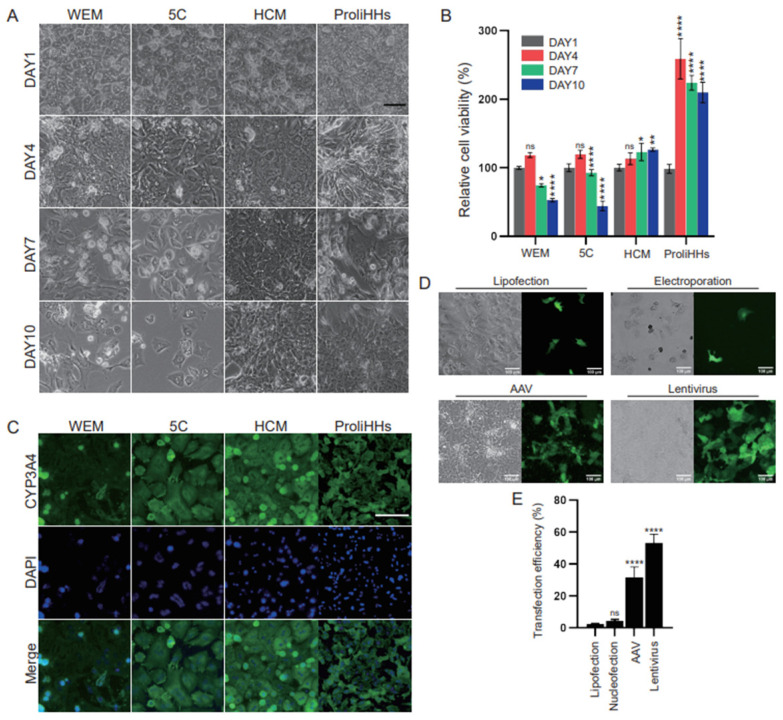
Comparison of cell culture and gene delivery efficiency in primary mouse hepatocytes. (**A**) Morphological changes in hepatocytes under four different culture media: WEM, 5C, HCM, and ProliHHs. Scale bar: 100 μm. (**B**) Cell viability under 4 culture media on day 1, day 4, day 7, and day 10. N = 3, * *p* < 0.05, ** *p* < 0.01, **** *p* < 0.0001. “ns” means not significant, *p* ≥ 0.05. (**C**) CYP3A4 immunofluorescence of the four media at day 10. Scale bar: 100 μm. (**D**) EGFP expression in hepatocytes with exogenous DNA delivered using four strategies: electroporation, Lipofection, AAV, and lentivirus. Scale bar: 100 μm. (**E**) The percentage of EGFP-positive hepatocytes using different DNA delivery strategies. N = 3, **** *p* < 0.0001, “ns” means not significant, *p* ≥ 0.05.

**Figure 2:**
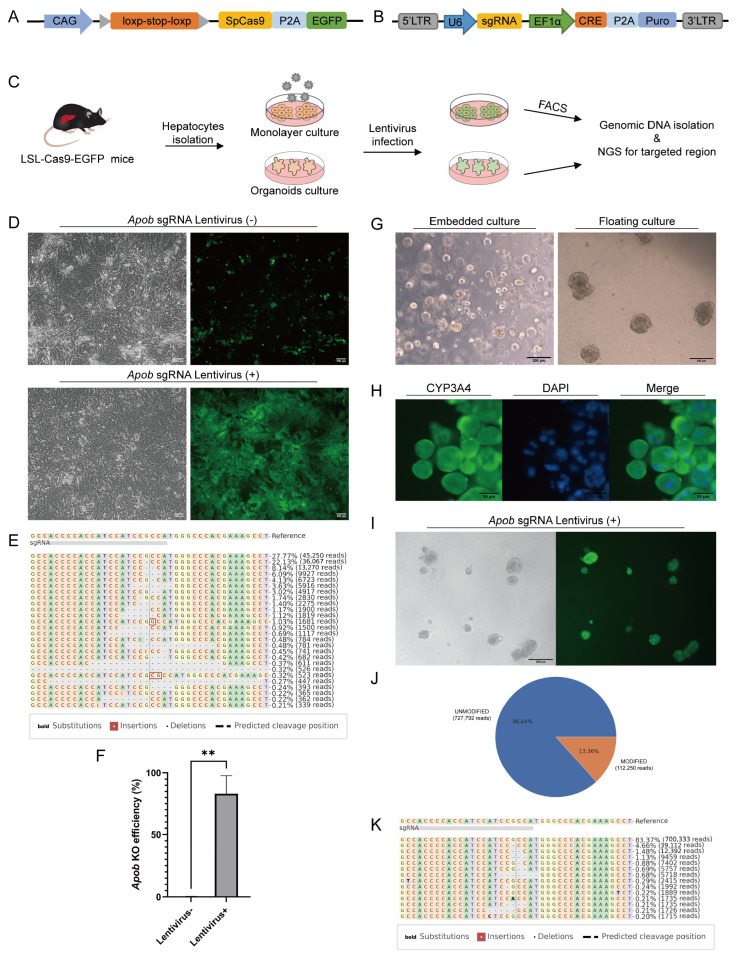
Lentiviral delivery strategy achieved genome editing of primary mouse hepatocytes cultured in both monolayer and organoid formats. (**A**) Schematic of LSL-Cas9-EGFP cassette knocked into the mouse Rosa26 locus. (**B**) Schematic of Cre-sgRNA lentiviral vector. (**C**) Schematic describing experimental workflow used to quantify single gene knockout efficiency via lentivirus. (**D**) EGFP expression in LSL-Cas9-EGFP hepatocytes transduced with CRE and sgRNA co-expressing lentivirus in monolayer cultures. (**E**) Distribution of indels and deletions around targeting site for the Apob sgRNA in monolayer cultures. (**F**) Apob knockout efficiency. N = 3, ** *p* < 0.01. (**G**) Organoids of primary mouse hepatocytes. Left: embedded cultured in Matrigel, right: floating cultured in 5% (vol/vol) Matrigel. Scale bar: 200 μm. (**H**) CYP3A4 immunofluorescence of mouse hepatocytes organoids. Scale bar: 50 μm. (**I**) EGFP expression of LSL-Cas9-EGFP hepatocytes transduced with CRE and sgRNA co-expressing lentivirus in organoid cultures. (**J**) NGS results showing Apob knockout efficiency in hepatocytes organoids. (**K**) Distribution of inserts and deletions around targeting site for the Apob sgRNA in organoid cultures.

**Figure 3:**
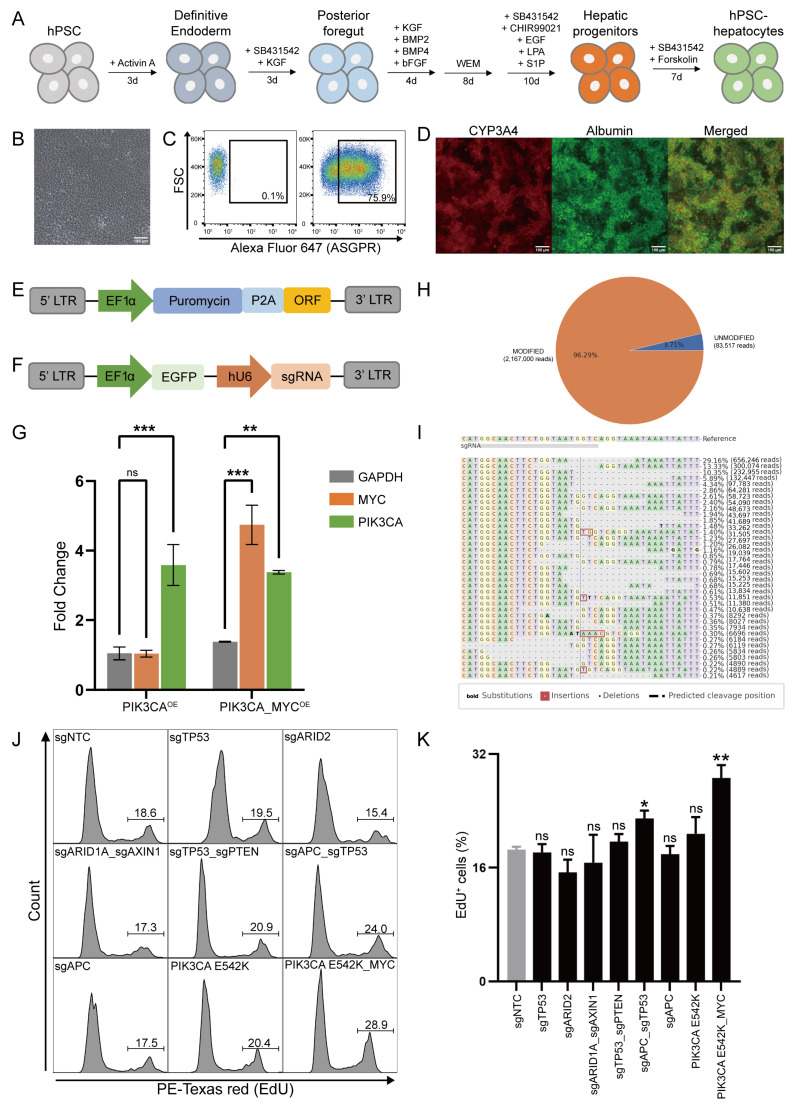
Genome editing of human pluripotent stem cell-derived hepatocytes. (**A**) Schematic describing the differentiation protocol from hPSCs to mature hepatocytes. (**B**) Morphology of hepatic progenitor cells differentiated from hPSCs. Scale bar: 100 μm. (**C**) Representative flow cytometric analysis showing the expression of ASGPR, a hepatic surface marker in hPSC-hepatocytes. (**D**) Co-immunofluorescence of CYP3A4 and Albumin in hPSC-hepatocytes. Scale bar: 100 μm. (**E**) Schematic of the oncogene ORF lentiviral vector. (**F**) Schematic of the sgRNA lentiviral vector. (**G**) Fold change in GAPDH, MYC, and PIK3CA mRNA between ORF overexpressed and control hPSC-hepatocytes. OE, overexpression. Two-way ANOVA analysis, N= 2, ** *p* < 0.01, *** *p* < 0.001, “ns” means not significant, *p* ≥ 0.05. (**H**) NGS results showing APC knockout efficiency in hPSC-hepatocytes. (**I**) Distribution of inserts and deletions around the targeting site for the APC sgRNA in hPSC-hepatocytes. (**J**) Flow cytometric analysis showing Edu incorporation 10 days after the introduction of oncogenic alterations into hepatic progenitor cells. (**K**) Percentage of Edu-positive cells. N = 3, * *p* < 0.05, ** *p* < 0.01, “ns” means not significant, *p* ≥ 0.05.

The data for Figure 1B and 1E were analyzed using two-way ANOVA and one-way ANOVA, respectively. The data for Figures 2E and 3G were analyzed using a *t*-test and two-way ANOVA, respectively. All statistical analyses were performed using GraphPad Prism 9 software. All experiments were performed in three replicates, except for the RNA sequencing experiments, which were performed in two replicates. All values are expressed as means ± SD. *p* values less than 0.05 were considered statistically significant. * *p* < 0.05, ** *p* < 0.01, *** *p* < 0.001, **** *p* < 0.0001. “ns” means not significant, *p* ≥ 0.05.

## 3. Results

### 3.1. Benchmarking Cell Culture and Gene Delivery Methods of Primary Mouse Adult Hepatocytes

To tackle the challenges of CRISPR genome editing in mature mouse hepatocytes, we first conducted a direct comparison of various hepatocyte culture methods to evaluate their effectiveness in maintaining cell viability and functionality. Alongside commercially available human hepatocyte culture media, William E medium (WEM) and HCM, we assessed two recently published protocols developed for human hepatocytes, termed “5C” [28] and “ProliHH” [29], to investigate their adaptability for application to mouse hepatocytes. HCM maintained the morphology and cell viability of primary mouse hepatocytes for at least 10 days, with a slight increase in relative cell viability observed on day 10 (Figure 1A,B). In contrast, hepatocytes in WEM and 5C media began to deteriorate starting on day 7, with 47% and 55% of the cells dying by day 10, respectively. Although our cell viability data showed that ProliHH medium supported hepatocyte expansion, the loss of the classical hexagonal shape and the appearance of a fibroblast-like morphology suggested that this medium concurrently induced cell dedifferentiation during the expansion process, consistent with previous reports [11] (Figure 1A,B). Immunofluorescence staining of the metabolic marker cytochrome P450 3A4 (CYP3A4) showed that the hepatocyte functionality was better preserved in HCM than WEM, 5C, or ProliHH media (Figure 1C). Therefore, HCM medium was superior to the other media for the subsequent genome editing efficiency validation in adult primary mouse hepatocytes.

The successful implementation of hepatocyte genome editing hinges on the effective intracellular delivery of genome-editing biomacromolecules. Therefore, we performed a systematic evaluation of DNA delivery methods, including electroporation, lipofectamine, adeno-associated viruses (AAVs), and lentivirus. Electroporation offers high transfection efficiency, particularly in challenging-to-transfect cells like primary cells, while lipofection is favored for its affordability and ease of use. Lentivirus is a widely used tool for CRISPR delivery, due to its stable genome integration, high transduction efficiency, and large cargo capacity. AAV is preferred for in vivo genome editing because of its high efficiency and specificity, especially in the liver, and its ability to achieve stable transduction in target cells. As evidenced by the expression of enhanced green fluorescent protein (EGFP), which was encoded with the plasmid DNA, all these methods successfully delivered the exogenous EGFP gene into mouse hepatocytes in vitro (Figure 1D). Electroporation showed slightly higher delivery efficacy compared to lipofectamine (4.42% vs. 2.47%), but it also reduced cell viability, leading to some hepatocytes being unable to attach to the plate (Figure 1D,E). Although the efficiencies of both electroporation and lipofectamine were generally low, the use of high-titer AAVs and lentivirus achieved up to 23 and 53% EGFP transduction, respectively (Figure 1D,E), without adversely affecting cell viability or morphology.

### 3.2. Genome Editing of Cultured Hepatocytes in Both Monolayer and Organoid Formats

As both lentiviral and AAV vectors have limited packaging capacity, we utilized a Cre-dependent Cas9 knockin mouse to overcome the packaging challenges associated with the large size of the Cas9 gene [30]. The expression of Cas9, as well as the self-cleaving P2A peptide linked EGFP, was interrupted by a loxP-stop-loxP (LSL) cassette and could be reactivated by the Cre recombinase (Figure 2A). We infected the hepatocytes isolated from the LSL-Cas9-EGFP transgenic mice with lentivirus encoding a sgRNA targeting Apob gene (Figure 2B,C). The high percentage of EGFP expression indicated the successful gene delivery and Cre-mediated recombination in hepatocytes (Figure 2D). Next-generation sequencing (NGS) analysis of the sgRNA targeted genome region in the EGFP+ hepatocytes showed the frequency of indels (insertion and deletion) was up to 80% (Figure 2E,F), suggesting the high efficiency of genome editing in the cultured hepatocytes.

In addition to the conventional monolayer culture format, the utilization of a 3D organoid culture system for primary hepatocytes has been reported [19,20], facilitating prolonged maintenance and moderate proliferation of mature hepatocytes. We implemented Hans Clever’s protocol to establish mouse primary hepatocyte organoids in both Matrigel-embedded culture and floating culture settings (Figure 2G). Maintenance of functionality in the organoid cultures was demonstrated by the expression of CYP3A4 (Figure 2H). Subsequently, we conducted genome editing in organoids by isolating hepatocytes from constitutive Cas9-expressing mice and delivering ApoB sgRNA via lentivirus (Figure 2B,I). Targeted NGS analysis revealed editing efficiencies in organoids ranging from 10 to 20% (Figure 2J,K), contrasting with the significantly higher editing efficiency observed in monolayer cultures.

### 3.3. Genome Editing of Human Pluripotent Stem Cell-Derived Hepatocytes for Modeling Tumorigenesis

Next, we conducted genome editing in human pluripotent stem cell (hPSC)-derived hepatocytes, as we lacked access to human primary hepatocytes. We improved our stepwise hPSC differentiation protocol to produce mature, functional hepatocytes (Figure 3A). Following 28 days of differentiation, we generated hepatic progenitor cells capable of proliferation (Figure 3B). Flow cytometric analysis revealed that 75.9% of the cells expressed the hepatic surface marker asialoglycoprotein receptor (ASGPR) (Figure 3C). After continuous culture of the FACS-enriched hepatic progenitor cells in hepatocyte mature medium for 7–10 days, immunofluorescence assays confirmed the co-expression of albumin (ALB) and cytochrome P450 3A4 (CYP3A4) in the hPSC-derived hepatocytes, indicating mature hepatocyte functionality and active phase-I metabolism (Figure 3D).

To recapitulate tumorigenic events in hPSC-derived hepatocytes, we employed a strategy involving the CRISPR-mediated knockout of tumor-suppressor genes coupled with the exogenous expression of mutated oncogenes. Based on mutation frequencies observed in The Cancer Genome Atlas (TCGA) liver hepatocellular carcinoma (LIHC) cohorts, we selected eight groups of single genes or combinations of tumorigenic genes for further study. These included knockout of three individual genes (ARID2, APC, TP53), double knockouts of three gene pairs (ARID1/AXIN1, APC/TP53, TP53/PTEN), exogenous expression of PI3KCA E452K mutant, and exogenous expression of both PI3KCA-E452K and c-MYC. Notably, loss-of-function mutations in TP53 are found in approximately 31% of hepatocellular carcinoma (HCC) patients [31]. Double-knockout of p53 and Pten has been shown to accelerate liver cancer development in mouse models [14]. In addition, co-occurring mutations of TP53 and APC are commonly observed across 20 types of solid tumors [32]. The WNT/β-catenin pathway is one of the major dysregulated pathways driving HCC, often through activating mutations in CTNNB1 and inactivating mutations in AXIN1 or APC [33]. Additionally, inactivating mutations in ARID1A or ARID2, which are key components of chromatin remodeling complexes, are recurrently altered in HCC [34]. Mutations in the PI3K/AKT/mTOR pathway are also identified in around 50% of HCC patients [34].

To initiate tumorigenesis, we introduced the CRISPR machinery and exogenous gene expression cassettes into the hPSC-derived hepatic progenitor cells using lentiviral delivery systems (Figure 3E,F), followed by continued differentiation to produce mature hepatocytes. The success of this genetic modification strategy was confirmed by the increased expression of the exogenously expressed genes in RNA sequencing data, and the high gene knockout efficiency (up to 96.29%) using a sgRNA targeting APC gene (Figure 3H,I). 

After 10 days, we assessed cell proliferation using an EdU assay (Figure 3J). Flow cytometric analysis demonstrated that cells with APC and TP53 double knockouts, as well as cells expressing exogenous PIK3CA-E542K mutant and MYC, exhibited significantly increased EdU incorporation (Figure 3K). Our findings underscored the critical roles of specific genetic alteration combinations in driving cancer growth. Thus, genome engineered hepatocytes can be used as a platform to evaluate the tumorigenic capacity of different gene aberrations.

### 3.4. Identification of Distinct Transcriptional Signatures in Genetically Modified Hepatocytes Carrying Various Genotypes

**Figure 4:**
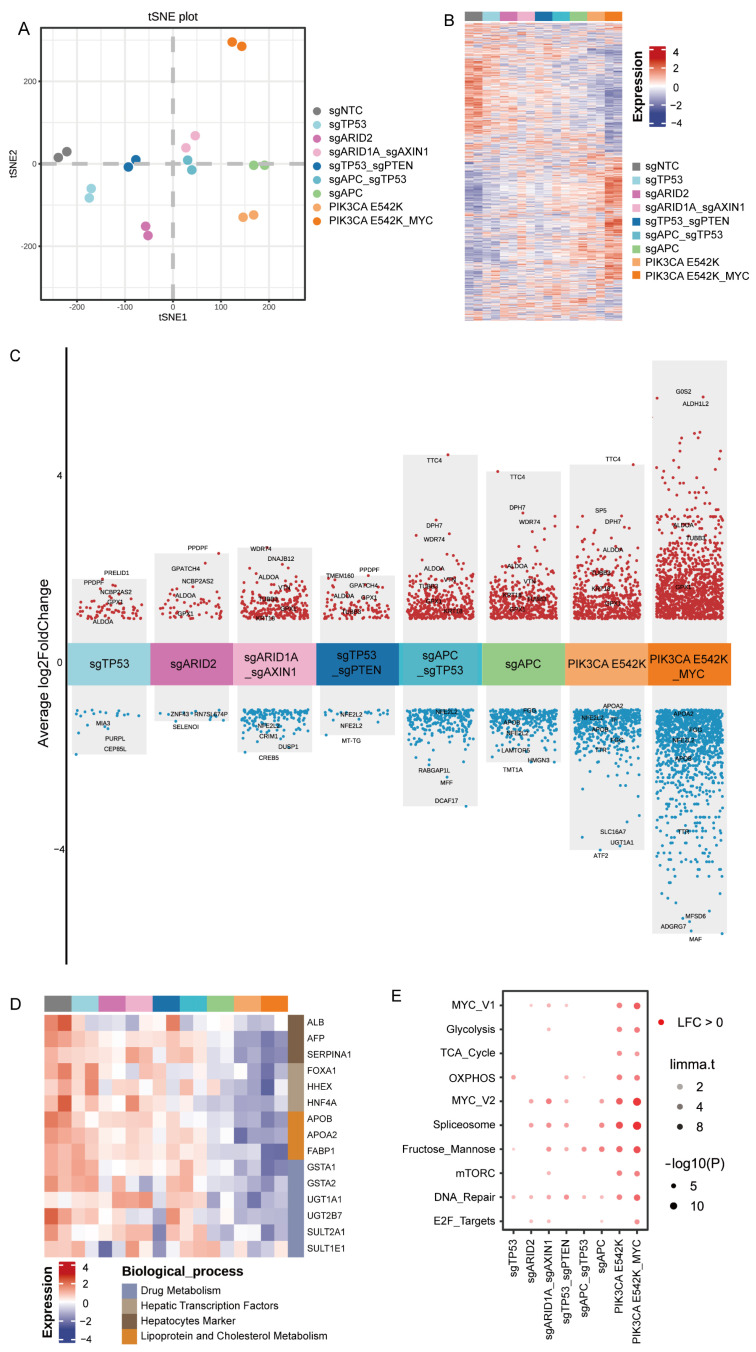
Transcriptional signatures of hPSC-derived hepatocytes with genome-engineered oncogenic alterations. (**A**) tSNE plot illustrating the transcriptomics of genome-engineered cells carrying various oncogenic alterations. (**B**) Heatmap displaying the top 2000 variable genes among all cell groups. (**C**) Volcano plot visualizing the differentially expressed genes (DEGs) for each cell group compared to cells transduced with the non-targeting control sgRNA (sgNTC). (**D**) Heatmap showing genes with significantly different expression levels, color-coded by gene functions. (**E**) Gene set variation analysis (GSVA) of the differentially expressed genes.

To test whether genome engineered hepatocytes resembled distinct tumor subtypes, we carried out transcriptome analysis of genetically manipulated cells isolated by FACS. The tSNE plot were used to visualize the overall relationship between the transcriptome of nine cell groups, each bearing distinct genotypes (Figure 4A). Notably, cells with identical genotypes clustered together, while those with different genotypes exhibited distinct transcriptional signatures (Figure 4B). Compared to the wild-type cells, the analysis of differential gene expression identified a significant number of upregulated and downregulated genes for each genotype. In particular, we observed that hepatocyte secretion proteins albumin, alpha-fetoprotein, and alpha-1-antitrypsin (encoded by ALB, AFP, and SERPINA1, respectively) were drastically downregulated in the PIK3CA-E542K, PIK3CA-E542K/c-MYC, and sgAPC groups, indicating these cell groups lost mature hepatic function after malignant transformation (Figure 4C,D). Similarly, decreased expressions of many hepatocyte functional markers, including hepatic transcription factors (FOXA1, HNF4A), drug metabolism genes (GSTA1, UGT1A1), and cholesterol metabolism genes (APOB, APOA2) were also observed in these groups (Figure 4D). Gene set variation analysis (GSVA) revealed that the hallmark of Myc targets, spliceosome pathway and DNA repair pathway, were activated by the introduction of oncogenic alteration. Interestingly, we observed increased activities of glycolysis, TCA cycles, oxidative phosphorylation, and fructose and mannose metabolism in the PIK3CA-E542K and PIK3CA-E542K/c-MYC groups, suggesting metabolic reprogramming occurred during the PI3KCA-driven cancer progression (Figure 4E). 

### 3.5. Discovery and Functional Analysis of Significant Gene Expression Modules in Response to Oncogenic Alterations 

**Figure 5:**
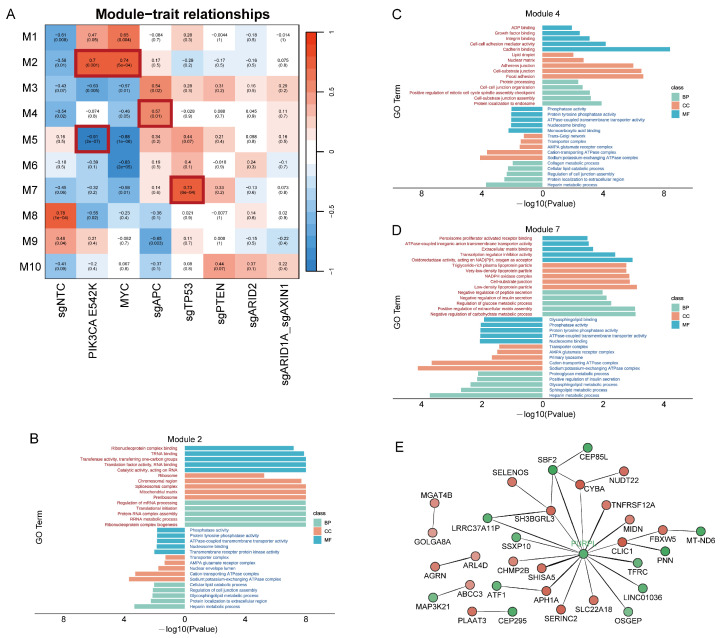
Identification of gene modules in response to individual oncogenic alterations in hPSC-derived hepatocytes. (**A**) The correlations between consensus module eigengenes and various oncogenic alterations. The corresponding *p*-values are shown in brackets. The color scale on the right shows correlations from −1 (blue) to 1 (red). M, module. (**B**–**D**) GO term enrichment analysis of genes in module 2 (**B**), module 4 (**C**) and module 7 (**D**). Left bars, pathways positively associated with the module. Right bars, terms negatively associated with the module. BP, biological process; CC, cellular component; MF, molecular function. (**E**) Genetic interaction network of top 30 interactions in module 7. The module membership scores, which represent the correlation between an individual gene and the module, are characterized by color of nodes from −1 (green) to 1 (red), and the weights of the interaction are characterized by the width of the edge.

These malignant cells, derived from the same parental cells and sharing the same genetic background, were genetically engineered to differ only in specific oncogenic alterations. This allowed us to investigate the gene expression modules responsive to each oncogenic alteration, without confounding effects of complicating genetic background noise. Using weighted gene co-expression network analysis (WGCNA), we identified 10 distinct modules from the transcriptomic profiles across nine sample groups, with module sizes ranging from 75 to 2064 genes. We hypothesized that each oncogenic alteration would regulate a unique downstream transcriptional program, whether present alone or in combination with other alterations. Consequently, we tested the correlation of gene expression modules with the individual manipulated genes, except for the ARID1A/AXIN1 double-knockout. Notably, module 2 was highly correlated with PI3KCA and MYC alterations, while module 4 and module 7 were significantly associated with APC and TP53 knockouts, respectively (Figure 5A). 

To further analyze the functions of genes in each module, we performed gene ontology (GO) term enrichment analysis. In particular, the module 2 genes were enriched in GO terms related to the RNA binding and activities, and depleted in hepatic functionality process such as cellular lipid catabolic, glycosphingolipid metabolic, and heparin metabolic (Figure 5B). These findings were consistent with our results showing downregulation of hepatic functionality in the PIK3CA and PIK3CA_MYC groups (Figure 4D). Module 4, responsive to the APC knockout, was linked with the functions involving cell junctions and adhesions (Figure 5C). Module 7 included pathways related with peptide secretion and lipoprotein particles (Figure 5D), consistent with previous reports that p53 is a known regulator of the lipid metabolism pathway [35].

To further explore the hub genes of the module linked to TP53 knockout, we computed the genetic interaction network of module 7 and visualized the top 30 gene–gene interactions. Interestingly, the long noncoding RNA (lncRNA) *PURPL* (p53 upregulated regulator of p53 levels) emerged as a center node in this network (Figure 5E). This finding aligns with previous observations of increased expression of *PURPL* in hepatocellular carcinoma patients. Additionally, *PURPL* is upregulated in response to DNA damage in a p53-dependent manner [28], and has been shown to participate in a regulatory feedback loop with p53 in colorectal cancer and liver cancer [36,37]. While the mechanism by which *PURPL* regulates numerous genes in the p53-linked module in hepatocellular carcinoma is highly intriguing, it is beyond the scope of the current study.

## 4. Discussion

In this study, we benchmarked two-dimensional (2D) and three-dimensional (3D) cell culture techniques along with various gene delivery methods, to optimize the genome editing of functional hepatocytes in vitro. Our findings indicated that up to 80% genome editing efficiency can be achieved in primary adult hepatocytes cultured in the monolayer format. Additionally, culturing hepatocytes as organoids yields moderate genome editing efficiency, while enabling the long-term maintenance of mature hepatocyte characteristics. Given the limited access to human primary hepatocytes due to ethical considerations, we established genome-engineered cellular models using hepatocytes derived from human pluripotent stem cells to recapitulate tumorigenesis.

We observed that the “5C” culture method, while effective in preserving the functionality of human hepatocytes, was not suitable for murine hepatocytes. This finding underscores the species differences between rodent and human liver, including variations in gene expression, metabolic capabilities, and disease progression [38]. The ProliHH method demonstrated efficacy in both human and mouse hepatocytes. However, its potential utility is hindered by the dedifferentiation of hepatocytes during culture. On the other hand, the organoid culture system strikes a balance between maintaining hepatocyte characteristics and genome editing efficiency; however, it is less convenient compared to the traditional monolayer culture format. 

Despite these limitations, human primary hepatocytes remain essential for studying liver physiology, signal transduction, metabolism, and hepatotoxicity in basic biomedical research and drug discovery, as mature hepatic functions cannot be fully reproduced by mouse models or hepatocytes derived from alternative sources. Therefore, successful genome editing of human primary hepatocytes in vitro is highly valuable for modeling liver diseases and conducting mechanistic studies. 

In addition to primary hepatocytes, hPSC-derived hepatocytes provide an alternative cell source for modeling tumorigenesis, with serval advantages. Firstly, human cells avoid significant species-specific differences, thereby representing the molecular mechanisms of human cancer initiation and progression more accurately than animal models. Secondly, the use of hPSC-derived cells circumvents ethical concerns associated with the use of primary human tissues. Thirdly, hPSC-derived hepatocytes ensure an unlimited, consistent, and reproducible cell population for experiments, as they can be generated under controlled conditions. Lastly, hPSC-derived hepatocytes can be easily cultured and genetically manipulated, enhancing the feasibility of experiments compared to primary hepatocytes. Overall, genome engineered hPSC-derived hepatocytes offer a versatile and scalable platform for modeling liver-related diseases, complementing research conducted using animal model primary hepatocytes.

Isogenic disease models, which differ exclusively in individual disease-causing genetic alterations, allow researchers to isolate and study the effect of specific genetic alterations, without the confounding variations common in non-isogenic models. This enhances the reliability and reproducibility of the experimental results, enabling more confident attribution of precise mechanisms to the genetic alterations under investigation. In this research, we developed a series of isogenic cancer models by introducing single or combinations of tumorigenic alterations into wild-type hPSC-derived hepatocytes. This strategy allowed us to shed light on early neoplastic events, discover key pathways and gene modules that alter during tumorigenesis, and identify potential therapeutic targets for hepatocellular carcinoma.

We observed a significant increase in cell proliferation and transcriptomic changes in the PIK3CA E542K overexpression groups, underscoring the potent effect of the activated PI3K/AKT pathway in driving malignant transformation. Notably, two key hallmarks of this transformation, loss of mature hepatic function and metabolic reprogramming, are predominantly influenced by the PI3K/AKT pathway. Previous studies have shown that IL-6, a growth factor upstream of the PI3K/AKT pathway, plays a crucial role in liver regeneration following partial hepatectomy in vivo [39], and in the expansion of cultured hepatocytes in vitro [40], both processes also involving hepatocyte dedifferentiation. These findings suggest that physiological liver regeneration and neoplastic transformation may share common pathways that regulate hepatocyte proliferation and dedifferentiation. 

It is intriguing that lncRNA *PURPL* emerged as a central node among the top 30 interactions within the TP53-related gene module. *PURPL* has been reported to be upregulated in liver cancer cell lines, although its exact mechanism remains unclear [36,37]. Additionally, the depletion of *PURPL* has been shown to elevate basal p53 levels by inhibiting the formation of the p53-MYBBP1A complex in colorectal cancer [41]. As the role of lncRNA *PURPL* is still not well understood, further research into its functions could provide new insights into HCC pathogenesis and potentially lead to novel tools for early diagnosis and treatment. 

Historically, generating a panel of isogenic cell models has been labor-intensive. Here, we overcome this challenge by developing an efficient and scalable platform using genome-engineered hPSC-derived hepatocytes. Our approach facilitates systematic analysis of single or combinations of genetic alterations in a single batch of experiments, thereby identifying both common and alteration-specific mechanisms of tumorigenesis.

## 5. Conclusions

Taken together, we achieved up to 80% single-gene editing efficiency in monolayer-cultured primary mouse hepatocytes using the lentivirus-based sgRNA delivery strategy, and up to 20% efficiency in cultured hepatocyte organoids. Utilizing hPSC-derived hepatocytes as an alternative to human hepatocytes, we investigated the roles of a spectrum of oncogenic alterations, both individually and in combination, in driving cancer outgrowth and altering transcriptomic phenotypes. We found that the PIK3CA E542K mutant, either alone or in combination with exogenous c-MYC, drastically promoted the malignant transformation of hepatocytes. Our findings highlighted the potential of genome-engineered hepatocytes in cancer modeling, offering new opportunities for high-efficiency and scalable gene function interrogation. 

## Figures and Tables

**Table 1:** sgRNA sequence.

Gene Name	sgRNA Sequence (5′-3′)
NTC	AAAAAGCTTCCGCCTGATGG
TP53	GGTGCCCTATGAGCCGCCTG
PTEN	ATTCTTCATACCAGGACCAG
APC	GGCAACTTCTGGTAATGGTC
ARID2	TGTGGTAGGAGTAAAACGGA
ARID1A	CAGCAGAACTCTCACGACCA
AXIN1	AGCCGGCATTGACATAATAG

Abbreviations: NTC, nontargeting control; TP53, tumor protein p53; PTEN, phosphatase and tensin homolog; APC, adenomatous polyposis coli; ARID2: AT-rich interactive domain-containing protein 2; ARID1A, AT-rich interactive domain 1A; AXIN1, Axis inhibition protein 1.

## Data Availability

Raw data are held by the authors and may be available upon reasonable request.
